# Utilisation of canteens offering healthy food choices as part of workplace health promotion in Germany

**DOI:** 10.25646/6401

**Published:** 2020-03-04

**Authors:** Susanne Jordan, Sophie Hermann, Anne Starker

**Affiliations:** 1 Robert Koch Institute, Berlin Department of Epidemiology and Health Monitoring; 2 Charité – Universitätsmedizin Berlin, Institute of Medical Sociology and Rehabilitation Science

**Keywords:** CANTEENS, SETTINGS-BASED PREVENTION, EMPLOYEES, DIET, HEALTH MONITORING

## Abstract

Ensuring that canteens offer employees healthy food choices is a settings-based measure of work-place health promotion. The German Health Update (GEDA 2014/2015-EHIS) surveyed the knowledge and use of canteens with healthy food choices by 18- to 64-year-old female and male employees. Over the previous twelve months, 64.6% of women and 66.2% of men had, where available, eaten at canteens with healthy food options at their place of work. These figures on canteen use decline with age. The most frequent use is by employees in the high education group. Women working full-time eat in canteens offering healthy food choices more often than women working part-time. No difference in relation to the number of hours worked is observed for men. Healthy food choices in canteens should continue to be promoted.

## Introduction

An appropriate diet and sufficient physical activity are important factors for health that contribute significantly to the prevention of non-communicable diseases such as type 2 diabetes, cardiovascular diseases and cancer [1]. The German Nutrition Society (DGE) has developed recommendations for a healthy diet which include consuming vegetables and fruit daily and consuming a needs-oriented amount of fish and dairy products [2]. However, these recommendations are often not put into practice or only partially [3]. For example, only around 40.4% of women and 23.9% of men eat vegetables every day [4]. The workplace provides an opportunity to promote a healthy diet, since a large proportion of the population works regularly. In 2017, around 71.5% of women and 78.9% of men aged 15 to 65 were in work [5].

The workplace health promotion can promote an appropriate diet through behaviour-related measures such as providing nutritional advice and information. However, settings-based measures to change the foods offered in canteens, cafeterias and vending machines are particularly effective [6]. The choice of food offered in canteens can even have an effect on employees with little interest in a healthy diet such as young men [7]. In 2017, around 19% of employees aged 14 and over ate at canteens and workplace cafeterias, while 13% went to bakeries or snack stalls and 4% went to restaurants [8].

Increasing the availability and accessibility of healthier products at workplace canteens can potentially lead people to make healthier food choices [9, 10]. One option is to display fruit and vegetables so they are easily visible, or to reduce the number of products that are less healthy. So-called ‘nudges’ can also unconsciously facilitate healthier food choices [9–11], for example, if healthier food is displayed attractively. To be successful, measures to promote healthy workplace diets must target both the setting and the individual behaviour [9].

**GEDA 2014/2015-EHIS**

**Data holder:** Robert Koch Institute

**Aims:** To provide reliable information about the population’s health status, health-related behaviour and health care in Germany, with the possibility of a European comparison

**Method:** Questionnaires completed on paper or online

**Population:** People aged 18 years and above with permanent residency in Germany

**Sampling:** Registry office sample; randomly selected individuals from 301 communities in Germany were invited to participate

**Participants:** 24,016 people (13,144 women; 10,872 men)

**Response rate:** 26.9%

**Study period:** November 2014–July 2015

More information in German is available at www.geda-studie.de

In the context of workplace health promotion, little is known about the number of canteens in Germany offering healthy food choices. To date, relevant data have been published by the German National Association of Statutory Health Insurance Funds (GKV-Spitzenverband) in their annual prevention reports [12] and from individual studies [13]. The survey GEDA 2014/2015-EHIS made it possible for the first time within health monitoring at the Robert Koch Institute to map the population’s use of canteens with healthy food options. Of particular interest was the extent to which socio-demographic factors and the number of hours worked influence canteen choices.

## Indicator

The indicator utilisation of canteens offering healthy food choices was populated in GEDA 2014/2015-EHIS using self-reported data from respondents completing paper-based or online questionnaires. The first question asked was ‘In the last 12 months, has your company had a canteen with healthy food offers (e.g. daily offer of vegetables, fresh salad and vegetarian dishes, regular offer of jacket or backed potatoes)?’ Respondents could answer with ‘Yes’, ‘No’, or ‘Don’t know’. If the answer was yes, the subsequent question was ‘Did you take up this offer?’, with the answers being ‘Yes’ or ‘No’. These questions were based on the study by Zok [13] and included additional examples from the DGE recommendations in ‘Eating at the Workplace and in Canteens’ [14]. The number of times people ate at canteens was not surveyed in GEDA 2014/2015-EHIS.

People were categorised as employed if they answered the question ‘Which life situation currently best applies to you?’ by stating that they were ‘working full-time, part-time or semi-retired’, were ‘marginally employed’, were having a ‘voluntary social/ecological/cultural year’, or were in ‘voluntary military service’ or in ‘federal volunteer service’ over the past twelve months. Employed people were divided into two groups depending on the number of hours they worked: ‘working part-time’ (which also included marginally-employed and semi-retired people) and ‘working full-time’. The results were stratified by sex, age group and education.

The analyses are based on data collected from 2,627 employed persons aged 18 to 64 who knew of a canteen at their workplace that offered healthy food options (1,244 women, 1,383 men). The present article reports on relative frequencies with 95% confidence intervals (95% CI). Confidence intervals were used to assess the precision of the estimated values, whereby broad confidence intervals indicate a greater statistical uncertainty of results. A significant difference is assumed if the p-value taking weighting and survey design into account is smaller than 0.05. The calculations were carried out using a weighting factor that corrects deviations from the population structure within the sample (as of 31 December 2014) with regard to sex, age, district type and education. The district type reflects the degree of urbanisation and corresponds to the regional distribution in Germany. The International Standard Classification of Education (ISCED), which is based on data on school and professional qualifications, was used to make the education data comparable [15]. The article German Health Update: New Data for Germany and Europe in issue 1/2017 of the Journal of Health Monitoring [16] contains a detailed description of the methodology applied.

## Results and discussion

The analyses of GEDA 2014/2015-EHIS show that around two-thirds of the women and men surveyed (64.6% and 66.2%, respectively) eat at a canteen that offers healthy food choices. No significant differences were observed between the sexes. The proportion of employees who take advantage of healthier options declines significantly in the 45- to 64-year-old age group. The difference between the youngest and the oldest age group is 10.3% for women and 8.4% for men. With the exception of the oldest group of women, employees of all ages in the high education group are more likely to eat at a canteen with healthy food choices than employees in the medium or low education group. This difference is not significant in the group of men aged 16 to 29 (Table 1).

**Figure 1:**
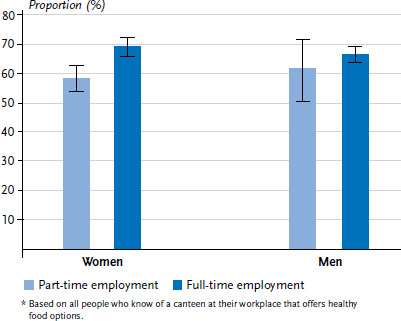
Relative frequencies of eating at a canteen with healthy food options by sex and number of hours worked (n=1,244 women, n=1,383 men)* Source: GEDA 2014/2015-EHIS

Significant differences were observed for women with regard to the number of hours worked. Women working full-time were more likely to state that they ate at a canteen with healthy food options than women working part-time (68.9% vs. 58.2%). No comparably significant differences were found for men (66.4% vs. 61.5%, Figure 1). The extent to which the specific working hours of women and men in part-time employment influences their choice of food cannot be deduced from the survey data.

The results of GEDA 2014/2015-EHIS essentially confirm the picture given by the sparse data on the promotion of workplace canteens offering healthy food choices, which forms part of the workplace health promotion in Germany. The 2008 Fehlzeiten-Report (report on absenteeism), for example, similarly indicates that around two-thirds of employees (66.9%) eat at canteens with healthy food options, and also finds a tendency towards lower rates in the older age groups [13]. An increase since then would have been expected, since the number of companies introducing measures to workplace health promotion in co-operation with statutory health insurance (SHI) has quadrupled to 17,672 over the past decade [12]. On the other hand, less than a third of the companies supported by SHI actually implemented a ‘healthy diet at work’ as part of efforts to workplace health promotion (for example 2014: 32%, 2017: 25%) [12, 17].

The lack of change in the use of canteens with healthy food options could therefore indicate that, as yet, only a few of the approximately 3,482,000 companies [18] have set their canteens up to offer healthy food choices as part of their workplace health promotion, possibly also because some do not have a canteen at all, for example where there are only a few employees.

In contrast to GEDA 2014/2015-EHIS, the Fehlzeiten-Report found higher usage for men (70.5%) than for women (62.2%) [13]. In GEDA 2014/2015-EHIS, slightly higher rates were recorded for women (64.6%) and slightly lower rates for men (66.2%). Further studies should show whether rates for women really have caught up with those of men in recent years. The more frequent use of healthy food options at canteens by employees with high levels of education is consistent with the general observation that groups with higher socioeconomic status are more likely to utilise preventive measures [19]. While the aim of settings-based preventive measures is, among other things, to help reduce social inequalities in health by modifying workplace conditions, canteens offering healthy food choices appear to be missing relevant target groups. This raises the question as to whether healthier choices at canteens are more expensive and therefore less attractive to low-income groups. These results suggest that further research should collect and evaluate information on the implementation of measures, the employment structure, the workplace and other influencing factors not provided by GEDA 2014/2015-EHIS. Any interpretation of GEDA data should consider the fact that these data are self-reported by employees, and may therefore be biased due to socially desirable responding or misinterpretation of questions.

The results presented on the use of canteens offering healthy food choices within as part of workplace health promotion show that, while Germany has begun to promote healthier food choices at canteens, in respect of equity in health, not all population groups are being reached equally. Quality standards for canteens should be further promoted [20], as should healthy food options in canteens. This would also help meet the increasing demand for healthy food options in the workplace [21].

## Key statements

- Two-thirds of male and female employees make healthy food choices if canteens provide healthy food options.
- The use of canteens with healthy food options declines with age.
- Employees in the high education group are the most likely to take advantage of these offers.
- Women working fulltime eat at canteens offering healthy food choices more frequently than women working part-time. There is no difference in relation to hours worked for men.

## Figures and Tables

**Table 1:** Relative frequencies of eating at a canteen with healthy food options over the past twelve months by sex, age and education status (n=1,244 women, n=1,383 men)* Source: GEDA 2014/2015-EHIS

Women			Men	
%		(95% CI)	%	(95% CI)
Total	64.6	(61.8–67.2)	66.2	(63.5–68.7)
**18–29 years**	70.6	(64.4**–**76.1)	69.9	(63.7**–**75.5)
Low education group	74.8	(53.2**–**88.5)	71.1	(52.7**–**84.5)
Medium education group	65.5	(57.3**–**72.8)	66.7	(58.3**–**74.2)
High education group	80.9	(70.9**–**88.1)	78.6	(68.1**–**86.4)
**30–44 years**	67.3	(61.9**–**72.2)	70.5	(66.3**–**74.5)
Low education group	58.6	(32.0**–**80.9)	57.8	(32.9**–**79.3)
Medium education group	61.7	(54.4**–**68.6)	66.8	(60.4**–**72.7)
High education group	77.3	(70.3**–**83.0)	76.4	(71.1**–**80.9)
**45–64 years**	60.3	(56.5**–**63.9)	61.5	(57.5**–**65.3)
Low education group	69.6	(57.9**–**79.3)	62.4	(49.1**–**74.0)
Medium education group	57.5	(52.3**–**62.6)	57.2	(50.7**–**63.4)
High education group	62.4	(57.1**–**67.4)	66.6	(62.2**–**70.7)

CI=Confidence interval

*Based on all people who know of a canteen at their workplace that offers healthy food options.
